# Modern and multidisciplinary care in polycythemia vera

**DOI:** 10.1007/s00277-026-06970-w

**Published:** 2026-04-18

**Authors:** Francesca Palandri, Chiara Sartor, Alessandro Pileri, Olga Addimanda, Christian Gagliardi, Michelangelo Sartori, Monica Benni, Maddalena Giannella, Federica Lo Dato, Elena Sabattini, Lorenzo Fiorino, Alessandra Dedola, Filippo Branzanti, Maria B. Rondinelli, Massimo Reta, Nazzareno Galiè, Pierluigi Viale, Benilde Cosmi, Michelangelo La Placa, Maria C. Morelli, Pier Luigi Zinzani

**Affiliations:** 1 Institute of Hematology “L. and A. Seràgnoli”, IRCCS Azienda Ospedaliero-Universitaria di Bologna, Via Massarenti 9, Bologna, 40138 Italy; 2 Dipartimento di Medicina Specialistica, Diagnostica e Sperimentale, Università di Bologna, Bologna, Italy; 3 Dermatology Unit, IRCCS Azienda Ospedaliero-Universitaria di Bologna, Bologna, Italy; 4 Rheumatology Unit, AUSL Bologna, Policlinico S. Orsola, AOU-IRCCS di Bologna, Bologna, Italy; 5 Cardiology Unit, IRCCS Azienda Ospedaliero-Universitaria di Bologna, Bologna, Italy; 6 Angiology and Blood Coagulation Unit, IRCCS Azienda Ospedaliero-Universitaria di Bologna, Bologna, Italy; 7 Department of Transfusion Medicine, AUSL, Bologna, Italy; 8 Infectious Diseases Unit, IRCCS Azienda Ospedaliero-Universitaria Di Bologna, Bologna, Italy; 9 Dipartimento di Scienze Mediche e Chirurgiche, Università di Bologna, Bologna, Italy; 10 Dipartimento di Psicologia, Università di Bologna, Bologna, Italy; 11 Haematopathology Unit, IRCCS Azienda Ospedaliero-Universitaria di Bologna, Bologna, Italy; 12 Dipartimento di Farmacia Clinica, Produzione e Ricerca, IRCCS Azienda Ospedaliero-Universitaria di Bologna, Bologna, Italy; 13 Internal Medicine Unit for the Treatment of Severe Organ Failure, IRCCS Azienda Ospedaliero-Universitaria di Bologna, Bologna, Italy

**Keywords:** Polycythemia vera, Transdisciplinary model, Cytoreductive treatment algorithm, Ropeginterferon alfa-2b, Ruxolitinib, Disease-modifying agents

## Abstract

Polycythemia Vera (PV) is a chronic, *BCR::ABL1*-negative, myeloproliferative neoplasm characterized by increased hematocrit and platelet/leukocyte counts, as well as by a high risk of thrombosis and progression to myelofibrosis and acute leukemia. The initial discovery of the *JAK2*^V617F^ driver mutation, followed by a more in-depth knowledge of the mechanisms that regulate inflammation and iron homeostasis, significantly advanced our understanding of PV pathophysiology, paving the way for a shift from symptom-focused vs. biology-focused treatments. PV management is complex and should be individualized, considering age, comorbidities, clinical presentation, disease course, and patient preferences. As such, optimal care requires a dynamic approach involving coordinated collaboration among healthcare professionals, in order to reduce complications, and enhance patients’ quality of life and outcomes. This narrative review explores principles and benefits of integrated care for PV patients, illustrated through two representative clinical cases. We propose that a transdisciplinary approach be adopted throughout the diagnostic and therapeutic continuum for PV to optimize patient outcomes. The review also proposes a novel cytoreductive treatment algorithm in PV going beyond the conventional risk stratification based on patient’s age and history of thrombosis but rather focusing on patient’s characteristics and treatment needs. Finally, we examine ongoing clinical studies and the latest results and insights on PV therapies (including the JAK inhibitor ruxolitinib, the next-generation mono-pegylated type I interferon ropeginterferon alfa-2b, the iron metabolism modifiers and other experimental approaches) that could reshape the standard of care in PV.

**Supplementary Information:**

The online version contains supplementary material available at 10.1007/s00277-026-06970-w.

## Introduction

Polycythemia vera (PV) is an acquired, chronic hematologic malignancy classified among *BCR::ABL*-negative myeloproliferative neoplasms (MPNs), alongside essential thrombocythemia (ET), primary myelofibrosis (PMF), pre-fibrotic MF and unclassifiable MPN (MPN-U), according to the 2022 International Consensus Classification (ICC) [1–3]. PV is invariably associated with a driver Janus Kinase 2 (*JAK2*) gene mutation, with canonical *JAK2*^V617F^ mutation in exon 14 occurring in > 95% of cases and non-canonical/atypical *JAK2* mutations in exons 12 to 15 in 3–5% of cases [4].

PV exhibits a slight male predominance and can present at any age, although the median age at diagnosis is 60 years, with incidence rising sharply in older populations [5]. The disease is characterized by uncontrolled erythrocytosis, increasing blood viscosity, and systemic symptoms such as pruritus, fatigue, headache, and visual disturbances, along with a heightened risk of thrombotic events (stroke, myocardial infarction, deep vein thrombosis). Concomitant proliferation of megakaryocytic and myeloid lineages may further elevate leukocyte and platelet counts, compounding thrombotic risk. PV also carries an increased risk of progression to post-PV myelofibrosis (PPV-MF) and acute myeloid leukemia (AML) [6–9].

### PV management: goals and key actions

PV management is based on age and thrombotic history, stratifying patients into low-risk (LR; <60 years, no prior thrombosis) and high-risk (HR; ≥60 years and/or previous thrombosis).

The primary goal is thrombosis prevention, a leading cause of morbidity and mortality in PV patients [9], that exhibit also a higher incidence of thromboembolic events (3.14% patients/year, %p-y) compared to both general population without (0.6%p-y) and with multiple cardiovascular (CV) risk factors (0.9%p-y) [10].

All patients should receive low-dose aspirin and phlebotomy, while cytoreductive therapy is reserved to HR subjects. However, additional disease features have recently been linked to increased thrombotic risk, indicating the need for refined risk models [11, 12].

Secondary goals include symptom control and quality-of-life (QoL) improvement, addressing pruritus, fatigue, fertility concerns, and minimizing treatment-related hematologic and non-hematologic toxicities, including secondary malignancies [13–15].

The third critical component is limiting disease progression to PPV-MF (cumulative risk between 4.9 and 6% at 10 years, 6–14% at 15 years and 26% at 20 years), or AML (cumulative incidence 2.3% at 10 years, 5.5% at 15 years and 7.9% at 20 years) [16].

**Fig. 1:**
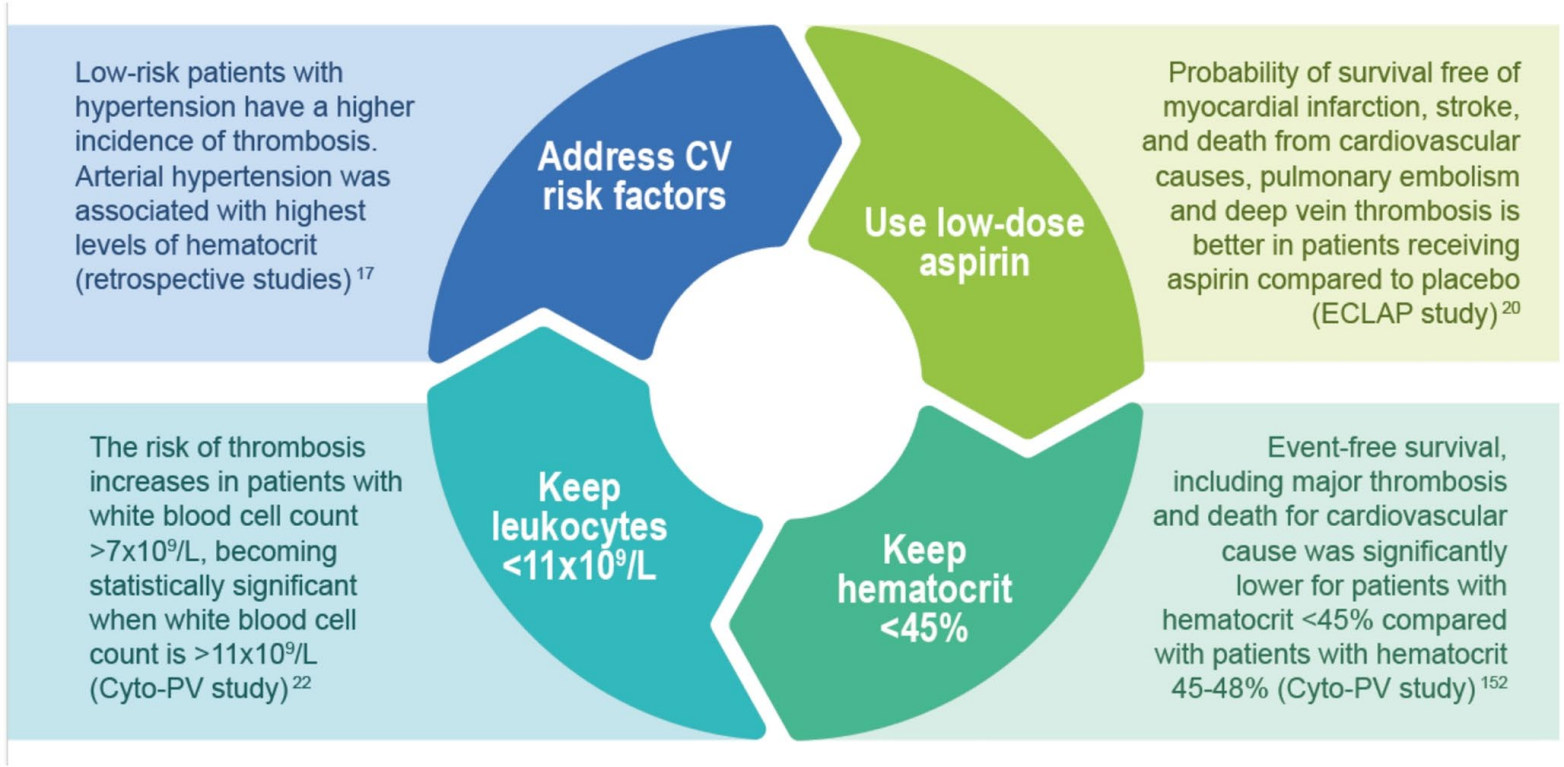
The four “must-dos” in polycythemia vera patients

To achieve these goals, careful selection of treatment type and timing, along with appropriate hematological and molecular disease control, may be relevant. We would recommend four key actions to optimize PV management across all patient groups (Fig. 1):

1. Managing CV risk factors: identification and correction of CV risk factors is crucial. CV risk factors comprise hypertension, diabetes mellitus, hyperlipidemias, tobacco use and overweight. A retrospective clinical study by Barbui et al. [17] revealed that low-risk PV patients who also presented with hypertension exhibited a notably increased incidence of thrombotic complications. Furthermore, hypertension in PV is frequently associated with elevated hematocrit levels. Therapeutic strategies aimed at reducing hematocrit allow alleviation of hypertension and confer a dual benefit by lowering the overall thrombotic risk. More recent data also include diabetes and hyperlipidemia as risk factors for arterial events [18, 19]. These findings underscore the importance of not underestimating the impact of all comorbid conditions, in all PV patients despite risk class.
2. Use of low-dose aspirin: this treatment has been shown to significantly improve PV outcomes. In particular, the ECLAP-study [20] showed that aspirin reduces the incidence of myocardial infarction, stroke, CV mortality and pulmonary embolism vs. placebo. Therefore, low-dose aspirin should be recommended for all patients. In case of intolerance, alternative agents should be prescribed.
3. Maintaining optimal hematocrit levels: maintaining hematocrit levels below 45% has emerged as a critical therapeutic target in the management of PV, with compelling evidence supporting its role in improving event-free survival. The landmark CYTO-PV trial, conducted by Marchioli et al. (2013), demonstrated that strict control of hematocrit (e.g., keeping it consistently below the 45% threshold) significantly lowers the incidence of major thrombotic events and reduces cardiovascular mortality compared to higher hematocrit thresholds [21, 22].
4. Maintaining leukocyte counts below the normal limits: in a sub-analysis of the CYTO-PV study, the risk of thrombosis increases along with the increase of leukocytosis and becomes statistically higher when the white blood cell (WBC) count exceeds 11 × 10^9^/L, making regular monitoring and management of WBC levels a critical aspect of treatment [23–25].

### Conventional cytoreduction in PV: which one, when and why

Hydroxyurea (HU) has long been the most frequently used first-line cytoreductive agent for thrombosis risk reduction in PV. As a ribonucleotide reductase inhibitor, HU impairs DNA synthesis, and proliferation of erythroid and myeloid lineages [26]. A phase 3 trial comparing HU to pegylated IFN-α (PEG) in treatment-naïve, HR ET/PV patients showed that both drugs are effective with similar thrombotic and progression rates [27]. Retrospective analyses from Spanish and Italian cohorts confirm the high efficacy of HU in PV, with overall response rates approaching 90%. Complete response (CR) was achieved in 24% of patients and partial response (PR) in 66%, highlighting HU as a cornerstone in first-line therapy [28–30]. Evidence from large retrospective cohorts indicates that HU significantly lowers the risk of recurrent arterial and venous thrombosis in MPN patients. Past studies have suggested a lower protective effect against venous recurrences following splanchnic vein thrombosis (SVT), indicating persistent vulnerability in this subgroup [31–33]. Recent data from a large cohort of 757 patients have redefined the role of cytoreduction, including HU, in SVT, showing a relevant reduction in SVT extension, recurrence and transjugular intrahepatic portosystemic shunt (TIPS) thrombosis [34].

Overall, a subset of HU-treated patients continues to show elevated thrombotic risk, phlebotomy-dependence or resistance, or develops HU intolerance [28, 29, 31–33, 35–42].

Ruxolitinib, a potent JAK1/2 inhibitor targeting aberrant signaling responsible for the proliferation of HSCs, is used as second-line in HU-resistant or -intolerant adults [43]. Its clinical efficacy in PV has been established in pivotal studies, including RESPONSE, RESPONSE-2, and MAJIC-PV [44–47]. Treatment progressively reduces *JAK2*^V617F^ variant allele frequency (VAF), a key predictor of long-term outcomes [48], and achieving any degree of molecular response during long-term therapy (median 8 years) is associated with improved overall, progression-free, thrombosis-free, myelofibrosis-free and event-free survival [49].

In a multinational real-world study, HR PV patients who switched from HU to ruxolitinib experienced clinical benefit in spleen size reduction and hematocrit control compared with non-switchers, the majority of whom remained on HU [50, 51]. Although such evidence, the PV-NET study revealed that 71.3% of patients did not switch to ruxolitinib despite suboptimal response, suggesting the need of better treatment management [33].

Of note, the investigational combination of ruxolitinib and pegylated interferon alfa-2a in newly diagnosed PV (COMBI II trial) resulted in high rates of hematologic and molecular responses, with a median *JAK2*^V617F^ VAF reduction from 47% to 7% and molecular remission achieved in 60% of patients [52].

Ropeginterferon alfa-2b (ropegIFNα2b), a monopegylated recombinant human interferon approved for PV without symptomatic splenomegaly, is increasingly favored as a first-line alternative to HU [53–55], particularly in younger patients [11], though it may cause mainly autoimmune and mood-related adverse effects [55].

IFNs are cytokines with immunomodulatory, antiviral and antineoplastic properties. IFN-α binds the IFNAR1/IFNAR2 receptor complex, activating JAK-STAT signaling and inducing IFN-stimulated genes. Signaling through PI3K and p38 MAPK pathways [56] promotes tumor suppressor, activation resulting in changes in gene expression and epigenetic modifications that affect cell growth, senescence, and the malignant cell phenotype [54, 57]. IFN-α specifically targets *JAK2*^V617F^ HSCs, particularly homozygous clones, suggesting that long-term exposure can alter PV outcome through a reduction of the *JAK2*^V617F^ allele burden [56, 58].

RopegIFNα2b’s extended half-life enables sustained JAK-STAT activation with less frequent dosing. Clinical studies, including PROUD-PV and CONTINUATION-PV, demonstrated its safety and efficacy in both LR and HR PV patients requiring cytoreduction [59, 60]. Rates of overall and complete hematologic response progressively increased in patients receiving ropegIFNα2b, whereas they declined in those treated with hydroxyurea, with the between‑group difference becoming statistically significant at later time points. Beginning at 24 months, patients treated with ropegIFNα2b exhibited significantly higher molecular response rates, which were also deeper and sustained for longer durations. These findings support the role of ropegIFNα2b as a disease-modifying agent [56]. In the Low-PV study, the addition of ropegIFNα2b to standard therapy (phlebotomy plus low-dose aspirin) improved hematocrit control (< 45%) [61]. A baseline neutrophil-to-lymphocyte ratio (NLR) ≥ 3.5 may identify LR PV patients eligible for early ropegIFNα2b therapy [62]. Higher dosing regimens can achieve faster complete hematologic response (CHR) but may be burdened by higher rates of adverse events [52, 63–67]. Common IFN-related toxicities include autoimmune diseases and mood alterations [68, 69].

Finally, the alkylating agent busulfan is considered a third-line option, as an alternative to ruxolitinib or ropegIFNα2b. Its use is mainly reserved for elderly patients and is typically administered in a cyclic schedule [70–72].

### The transdisciplinary approach to PV treatment

PV management is inherently complex and should be individualized, considering age, comorbidities, clinical presentation, disease course, and psychosocial, logistical, and health literacy factors, alongside patient preferences. Treatment challenges include cytoreductive therapy toxicity, caregiver dependence, phlebotomy burden, and risk of disease-related complications, highlighting the need to involve multiple healthcare professionals [73].

Care integration can evolve from parallel professional involvement (multidisciplinary) to coordinated collaboration (interdisciplinary), and ultimately to a fully integrated, patient-centered model (transdisciplinary). In the multidisciplinary setting, professionals contribute independently, often with limited cross-disciplinary exchange. Transdisciplinary care reflects the highest level of integration, where team members transcend disciplinary boundaries, adopt common conceptual frameworks, and jointly address complex clinical challenges through collective decision-making. This model enhances therapeutic coherence and fosters a holistic understanding of patient needs, values, and trajectories (Fig. 1S). Transdisciplinary care is particularly relevant for managing multifaceted conditions such as MPNs, where biological, psychosocial, and systemic factors converge and demand unified, adaptive responses [74].

The following section illustrates the implementation of transdisciplinary care through two representative PV clinical cases.

## Clinical case 1 - a difficult diagnosis of polycythemia vera

A 40-year-old woman presented to the emergency department with melena and three-days history of right upper quadrant abdominal pain and nausea. Her history included suboptimal controlled hypertension, mild dyslipidemia, and overweight (BMI 28), with no alcohol or tobacco use.

Physical examination revealed mild ascites, lower limb edema, and increased tenderness in the upper abdominal quadrants. Laboratory tests showed: hemoglobin 14.8 g/dL, hematocrit 46%, mean corpuscular volume (MCV) 71 fL, WBC 13.4 × 10⁹/L (neutrophils 8.2 × 10⁹/L, lymphocytes 2.5 × 10⁹/L – NLR 3.28), platelets 356 × 10⁹/L, elevated transaminases and direct hyperbilirubinemia, hypoalbuminemia, and low ferritin; coagulation parameters were normal.

Doppler ultrasonography (US) showed a patent inferior vena cava, thrombosis of the right portal vein branch, absent hepatic vein flow, and splenomegaly (spleen diameter: 18 cm). Abdominopelvic computed tomography (CT) and magnetic resonance imaging (MRI) identified moderate ascites, hepatosplenomegaly with caudate lobe hypertrophy, and abnormal hepatic perfusion. Venous thrombosis was confirmed, establishing the diagnosis of Budd-Chiari syndrome (BCS).

Consultations with specialists in thrombosis and hemostasis and hepatologists led to initiation of anticoagulation with low-molecular-weight heparin (LMWH) and diuretic therapy. A TIPS was performed, which promptly reduced portal pressure.

Work-up for hypercoagulable states, neoplastic, and autoimmune disorders was unremarkable. Hematological assessment identified *JAK2*^V617F^ mutation with a VAF of 14%. Bone marrow biopsy showed a hypercellular bone marrow with panmyeloisis consistent with PV. Low iron markers explained normal hemoglobin levels, that initially delayed PV suspicion.

### The transdisciplinary approach to PV diagnosis

PV diagnostic criteria were recently reviewed within the 2022 WHO guidelines [3] and ICC [1, 2]. Although seemingly straightforward, recognition of PV outside specialist centers is often limited, resulting in potential delays in diagnosis and treatment, negatively impacting patient outcomes [75, 76]. When presenting with a major thrombotic event, initial investigations typically prioritize exclusion of non-hematologic prothrombotic causes [13]. Early implementation of a transdisciplinary approach is crucial for timely recognition of PV in splanchnic thrombosis, as recognized by the Baveno VII consensus on portal hypertension [77].

Table 1 summarizes the updated WHO diagnostic criteria and emphasizes the transdisciplinary integration of clinical, pathological, molecular, and multidisciplinary perspectives for optimal PV diagnosis.

**Table 1:** Updated WHO/ICC 2022 diagnostic work-up for polycythemia vera with transdisciplinary integration

Diagnostic domain	Criteria	Discipline(s) involved	Notes/Integration points
Major Criteria			
1. Hematology values	Hb > 16.5/16.0 g/dL (men/women) or Hct > 49%/48% (men/women) or increased red cell mass	Hematologist, Laboratory medicine	Consider confounding factors (iron deficiency, hemodilution, B12 and B9 vitamins deficiency, splenomegaly)
2. Histopathology	Hypercellularity with trilineage growth (panmyelosis*)	Hematopathologist	Required to distinguish PV from other MPNs, MDS, non-clonal disorders
3. Molecular clonal markers	Presence of *JAK2*^V617F^ or *JAK2* exon 12–15 mutation	Molecular biologist, Hematologist	Confirms clonality; use highly sensitive assays for *JAK2* V617F (sensitivity < 1%); if negative, consider noncanonical *JAK2* (3–5%) [3]
Minor Criterion			
1. Laboratory values	Low serum EPO level	Laboratory medicine	Supports diagnosis in context of other findings
Additional integration			
1. Additional molecular clonal markers	Assessment for subclonal myeloid mutations	Molecular biologist, Hematologist	Assessed by sensitive NGS; myeloid-associated mutations detected in > 50% of cases (e.g., *ASXL1*,* EZH2*,* IDH1*,* IDH2*,* SF3B1*,* SRSF2*,* and TET2*) [1, 3]
2. Exclusion of secondary causes	Rule out secondary erythrocytosis**	Hematologist, Molecular Biologist, Internist, Radiologist	Supports diagnosis in the context of other findings: • Chest X-ray and spirometry to exclude lung disorders • NGS analysis for congenital causes of erythrocytosis • Polysomnography to exclude Obstructive Sleep Apnea Syndrome (OSAS)
3. Thrombotic and hemorrhagic risk assessment	Evaluation for non-hematologic prothrombotic or pro-hemorrhagic conditions	Hematologist, Specialists in thrombosis and hemostasis, Rheumatologist, Hepatologist, Cardiologist, Nutrition Medicine Specialist	Essential steps for accurate antithrombotic prophylaxis: • Exclude antiphospholipid syndrome • Screen for inherited thrombophilia • Rule out acquired von Willebrand disease • Evaluate modifiable cardiovascular risk factors
4. Symptoms evaluation	Evaluation of psychological distress, health literacy, PV-related symptoms	Hematologist, Transfusion medicine specialist, Clinical research nurse, Onco-psycologist	Essential for quantifying disease burden: • MPN10-TSS (Myeloproliferative Neoplasm Symptom Assessment Form Total Symptom Score) [76] • Distress Thermometer (DT) [78]

*EPO* erythropoietin, *Hb* hemoglobin, *Hct* hematocrit, *JAK2* Janus kinase 2, *MPN* myeloproliferative neoplasms, *PV* polycythemia vera, *MDS* myelodysplastic syndromes *Panmyelosis: proliferation of erythroid, granulocytic, and megakaryocytic lineages; **Secondary erythrocytosis: e.g., hypoxia, EPO-secreting tumors, drugs, congenital erythrocytosis

#### The role of the hematologist

Erythrocytosis may be masked by factors determining hypoxia (e.g., smoking, lung disorders, obstructive sleep apnea), altered erythropoietin (EPO) production, or medications such as sodium-glucose cotransporter 2 (SGLT-2) inhibitors, diuretics, or testosterone, that may provoke secondary erythrocytosis [79]. In patients with SVT, hemodilution and hypersplenism from portal hypertension often obscure laboratory and clinical findings of MPN, including increased blood values and splenomegaly. Bleeding from gastroesophageal varices and iron deficiency may further conceal erythrocytosis. A comprehensive evaluation of complete blood counts, pre-phlebotomy EPO levels, molecular mutations, and exclusion of secondary causes of polyglobulia require hematologic expertise.

#### The role of the hepatologist

SVT occurs in 0.7 to 2.7 per 100,000 patient-years [80], much less frequent than usual-site venous thromboembolism (138 per 100,000 patient-years). SVT may manifest as portal, splenic, or mesenteric vein thrombosis, or BCS, and can be associated with local disorders (abdominal cancer, liver cirrhosis, intra-abdominal inflammation, surgery) or systemic conditions (hormonal treatment, thrombophilic conditions). Portal vein thrombosis and BCSs are 2,000 and 10,000-fold more frequent in MPN patients than in the general population. In non-cirrhotic SVT, a MPN should be investigated, being diagnosed in 30–40% of cases [81, 82], with PV being the most frequent. MPN-SVT shows a predilection for women under 45 years and usually presents within the first year of MPN diagnosis.

Work-up for primary SVT includes evaluation of prothrombotic factors and systemic diseases, but risk factor identification should not replace comprehensive assessment, as up to 30% of cases are triggered by multiple pro-thrombotic factors [83]. Current recommendations emphasize prompt hematological evaluation and testing for the *JAK2*^V617F^ mutation in all patients [77].

In patients with SVT, the hepatologist plays a pivotal role not only in initiating anticoagulation but also in determining the optimal timing for TIPS placement when first-line medical therapy fails. In BCS and portal vein thrombosis associated with MPNs such as PV, early recognition of inadequate response to anticoagulation is essential to prevent liver failure, variceal bleeding, or intestinal ischemia and to allow timely escalation to interventional procedures, including TIPS. In this setting, TIPS is increasingly used to control portal hypertension and preserve portal venous inflow; however, its indication and timing require careful patient selection and coordinated follow-up between hepatologists and hematologists [82, 84–86].

#### The role of the molecular biologist

PV is driven by gain-of-function mutations in the *JAK2* gene (most commonly *JAK2*^V617F^ in exon 14, found in over 95% of cases, with less frequent mutations in exons 12 to 15 occurring in 3–5%) [4, 87]. *JAK2* mutations constitutively activate the JAK-STAT signaling, driving uncontrolled HSC proliferation, especially of the erythroid lineage [88]. Non-canonical *JAK2* mutations can also be detected in *JAK2*^V617F^-negative patients and may support the diagnosis of PV [89].

In case of undetectable *JAK2* mutations, but strong suspicion for PV, additional molecular evaluations are required, including the exclusion of MPL and calreticulin mutations. Recent data highlight the possibility of double or triple-driver mutations in MPNs including PV, supporting the testing for molecular clonality beyond JAK2 mutations [90]. Using next generation sequencing (NGS) techniques, over 50% of patients harbor additional non-*JAK2* mutations, most commonly *TET2* (18%) and *ASXL1* (15%) [8], which carry prognostic significance and could inform PV risk models [87, 91–93].

Early NGS in non-cirrhotic, non-tumoral portal vein thrombosis can improve diagnostic accuracy, particularly in “triple-negative” cases, and guide prognosis and therapy [82, 94, 95].

#### The role of the hematopathologist

*JAK2* mutations confirm clonality, abrogating secondary or spurious erythrocytosis, but cannot alone distinguish PV from other MPNs; a combination of clinical, histopathological and molecular criteria is required. Bone marrow typically shows hypercellularity, panmyelosis with relative elevation of erythropoiesis, clusters of polymorphous megakaryocytes, absent stromal iron, and minimal or no fibrosis.

Although histology is a key diagnostic tool, subjectivity and overlapping features among MPNs subtypes necessitate an experienced hematopathologist. Histological evaluation also provides prognostic information, and future integration of deep learning models, including artificially enhanced cytological evaluation, with clinical parameters could further enhance diagnostic accuracy [82, 96–98].

### The transdisciplinary approach to PV front-line therapy

**Fig. 2:**
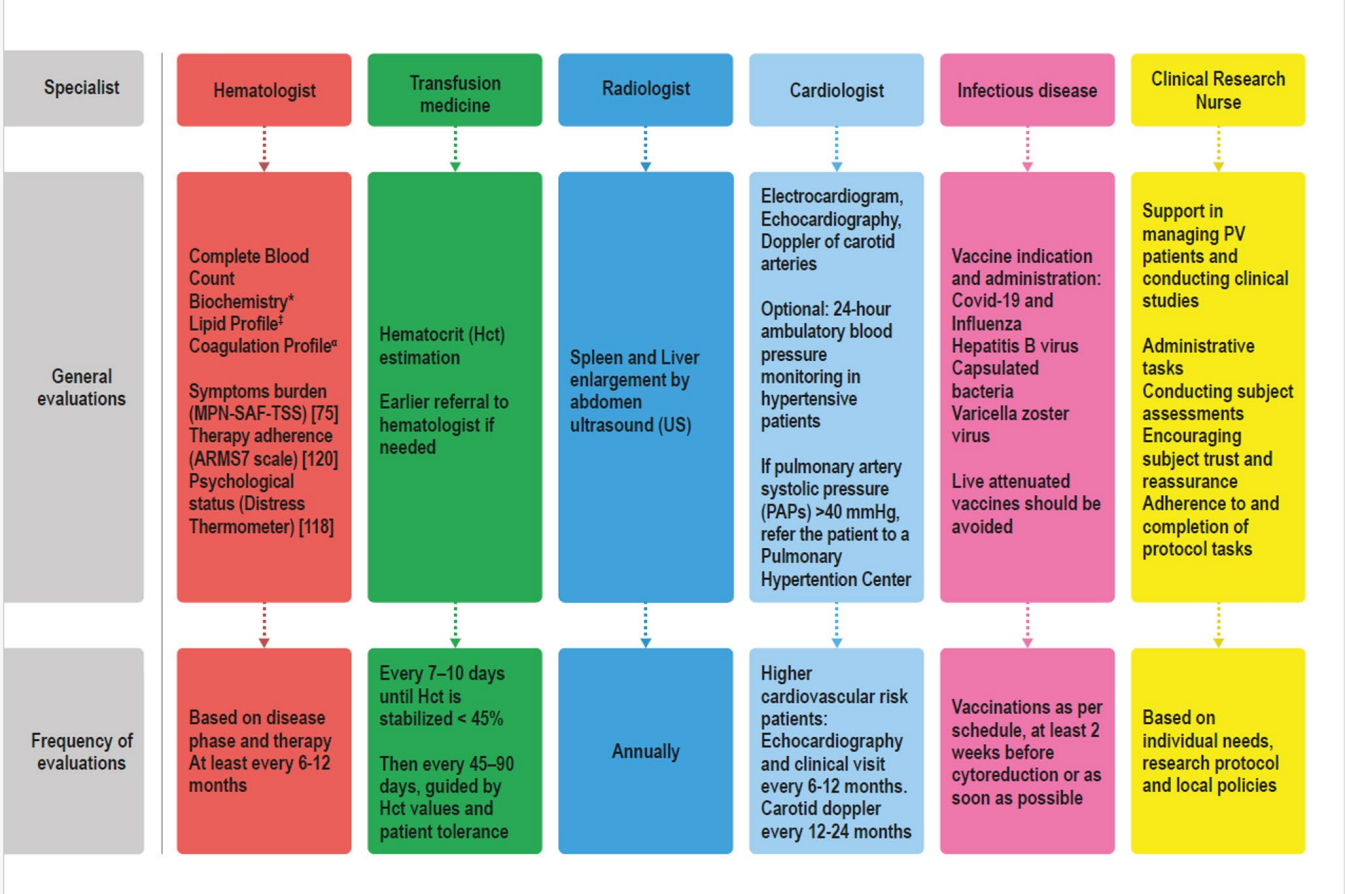
Multidisciplinary assessment of all patients with polycythemia vera. *Biochemistry: Glucose, HbA1c, Creatinine, Blood Urea Nitrogen, uric acid, Sodium, Potassium, Total and Fractionated Bilirubin, Glutamate Oxaloacetate and Pyruvate Transaminase (GOT/GPT); Gamma-Glutamyl Transferase (GGT), Alkaline Phosphatase (ALP), Albumin, total serum protein, Erythrocyte Sedimentation Rate (ESR); C-Reactive Protein (CRP). Lipid Profile: Total/LDL Cholesterol, Triglycerides. ^α^Coagulation Profile: Prothrombin Time (PT), Activated Partial Thromboplastin Time (aPTT), Antithrombin III activity, Fibrinogen. Definitions: Very High cardiovascular risk: history of thrombosis; High Risk: age > 60 years. Abbreviations: MPN-SAF TSS: Myeloproliferative Neoplasms Symptoms Assessment Form Total Symptoms Score; ARMS: Adherence to Refills and Medications Scale; PV: Polycythemia Vera

**Fig. 3:**
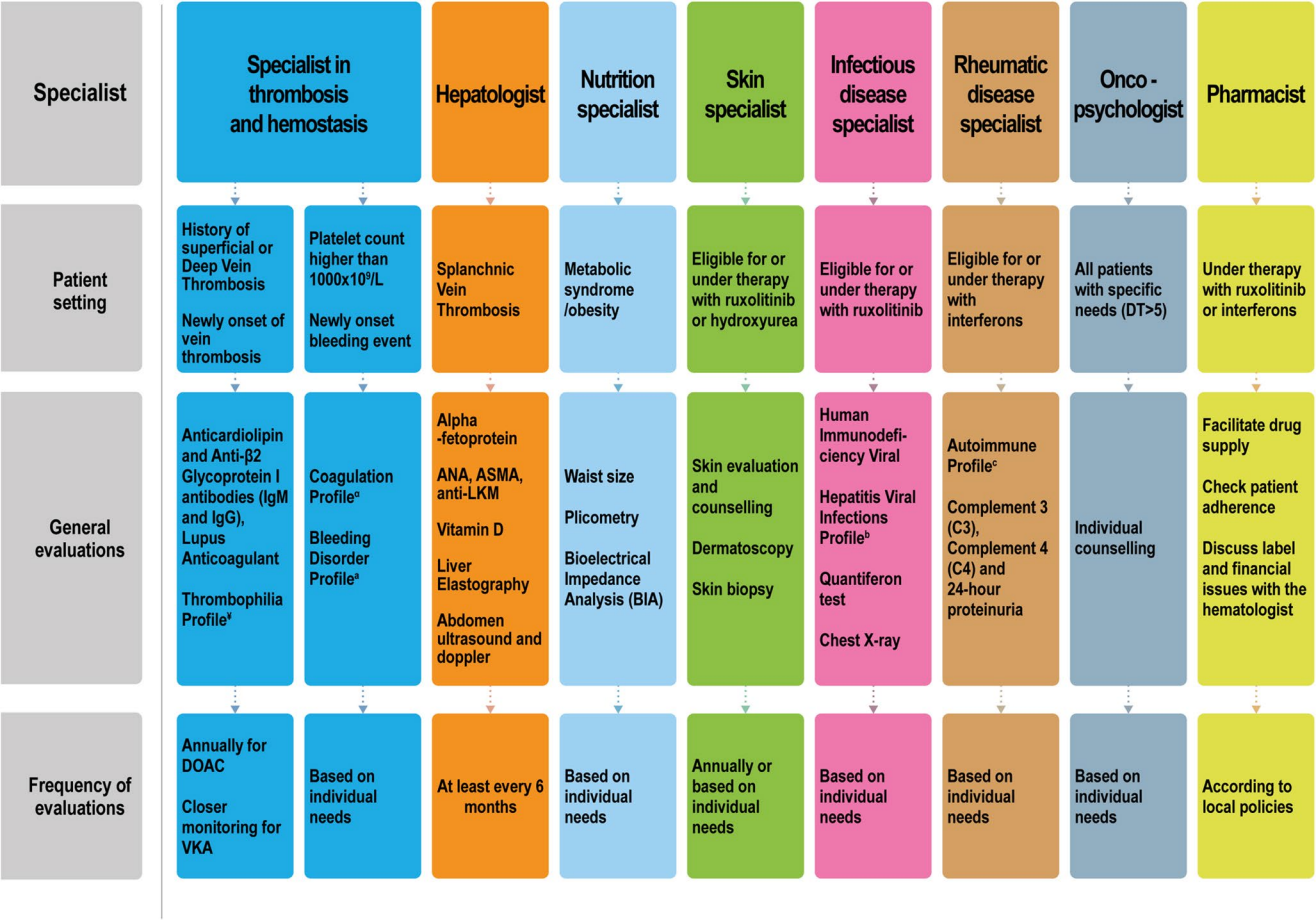
Multidisciplinary assessments for patients with polycythemia vera in specific clinical contexts. ^α^Coagulation Profile: Prothrombin Time (PT), Activated Partial Thromboplastin Time (aPTT), Antithrombin III activity, Fibrinogen. ^¥^Thrombophilia Profile: Protein C activity, Protein S activity, Activated Protein C resistance, G20210A mutation, Factor VIII (high range), Homocysteine. ^a^Bleeding Disorder Profile: von Willebrand factor, Platelet Function Assay (PFA-100) (Collagen-Epinephrine), PFA-100 (Collagen-ADP, adenosine diphosphate), Blood group, factor VIII. ^b^Hepatitis Viral Infections Profile: anti-HCV (Anti-Hepatitis C Virus), HBsAg (Hepatitis B Surface Antigen), anti-HBc (Anti-Hepatitis B Core Antibodies), anti-HBs (Anti-Hepatitis B Surface Antibodies), anti-HEV (Anti-Hepatitis E Virus) ^c^Autoimmune Profile: ANA reflex, RF (Rheumatoid Factor), ACPA (Anti-Citrullinated Protein Antiboides), LAC (Lupus Anticoagulant), anti-beta2 Glycoprotein I IgG and IgM, anticardiolipin IgG and IgM, C3 and C4. Definitions: Metabolic syndrome: Waist circumference, ≥ 102 cm (≥ 40 inches) for men and ≥ 88 cm (≥ 35 inches) for women; Fasting blood glucose, ≥ 100 mg/dL (≥ 5.6 mmol/L), or the use of hypoglycemic therapy; Blood pressure, ≥ 130/85 mmHg, or Treatment for Hypertension; Triglycerides, ≥ 150 mg/dL (≥ 1.7 mmol/L), or the use of Lipid-lowering therapy; HDL cholesterol, < 40 mg/dL (< 1.04 mmol/L) for men and < 50 mg/dL (< 1.29 mmol/L) for women, or the use of anti-dyslipidemia treatment. Abbreviations: DOAC: Direct Oral Anticoagulants; VKA: Vitamin K antagonists; ANA: Antinuclear Antibodies; ASMA: Anti-Smooth Muscle Antibodies; anti-LKM, Anti-Liver Kidney Microsomal Antibodies; DT: Distress Thermometer [78]

After hematologist counseling, the patient started phlebotomies and cytoreductive therapy with HU. During hospitalization, she remained hemodynamically stable, with progressive clinical and laboratory improvement, and was subsequently discharged and referred to a transdisciplinary group for further consultations and follow-up (Figs. 2 and 3). Treatment monitoring involved:

*- Specialist in thrombosis and hemostasis:* for thrombosis assessment and anticoagulation management. The standard initial treatment of SVT consists of low-molecular-weight-heparin (LMWH). Early initiation of anticoagulation increases the rates of vessel recanalization and improves prognosis [99]. Moreover, LMWH allows dose reductions in case of severe thrombocytopenia. Vitamin K antagonists (VKA) or direct oral anticoagulants (DOACs) can be considered for the long-term treatment of SVT. In this case the patient was a candidate for long-term anticoagulation. Based on patient preference and the growing evidence supporting DOAC use in non-cirrhotic patients, a DOAC was started [100–105].

*- Transfusion medicine specialist:* for phlebotomy management. Phlebotomies were initially performed every 10 days to rapidly lower hematocrit. The target hematocrit was set at 42% due to SVT and hyper-viscosity symptoms [80, 105–107]. Phlebotomy should begin as soon as possible after PV diagnosis. During induction, the regimen should consider a person’s weight and remove 300-450 mL of blood every other day or twice week until target hematocrit is achieved. In the maintenance phase, the intervals are adapted according to hematological response and patient tolerance. Potential complications include iron deficiency, thrombocytosis, and intolerance [106].

*- Hematologist.* For cytoreduction management. HU was started at 500 mg daily with good clinical tolerance. During the first 3 months, complete blood count and liver enzymes were evaluated monthly, followed by a clinical visit with full laboratory exam. Treatment requires careful monitoring of platelet values to minimize bleeding risk, in patients already at risk for portal hypertension and ongoing anticoagulation therapy. HU is the most used agent, with ruxolitinib emerging as additional therapeutic option in case of HU resistance/intolerance [34, 108]. Interferons have also been used [109, 110].

*- Cardiologist: *for cardiovascular risk assessment and therapy optimization.

Anti-hypertensive therapy was modified to achieve optimal hypertension control, and statins introduced to correct the lipidic profile, with annual follow-up scheduled.

CV risk factors (CVRF) significantly impact thrombotic risk and survival in PV [111]. All PV patients should undergo evaluation of modifiable CVFR, as PV adds a CV burden comparable to diabetes and chronic kidney disease. Based on CVRF anamnesis, comprehensive of 12-lead electrocardiogram (ECG), transthoracic echocardiogram, Doppler US of the supra-aortic trunks and renal function assessment [19], patients are stratified in low, moderate, high and very high risk. CVRF assessment should be performed on an annual basis [112, 113]. Although no PV-specific dyslipidemia guidelines exist, aggressive management is advised; an LDL-C target < 70 mg/dL, as in high CVRF patients, seems reasonable [114]. Also, smoking negatively affects response and outcomes in MPN patients [115]. Patients should be strongly encouraged to cease all forms of smoking, including vaping, as part of comprehensive cardiovascular risk reduction.

*- Nutritionist:* for implementation of dietary intervention.

Life-style modifications are important for CV risk reduction. PV patients should be instructed to follow a correct diet, engage in regular physical activity, and control body weight.

*- Dermatologist: *for annual full-body skin review.

HU can cause dermatological toxicities, from common effects such as hyperpigmentation of skin and nails, to more severe including leg ulcers, dermatomyositis-like eruptions and non-melanoma skin cancer (NMSC). For the latter, specialistic monitoring is advised, particularly in HU-exposed patients with additional risk factors such as age, prolonged sunlight exposure, higher dose and long-term HU use [38, 116]. Full-body skin checks are recommended at least annually [117], and patients should avoid sun exposure, use high protection sunscreens, and promptly report new lesions. In patients with precancerous or cancerous lesions, dermatologic follow-up frequency should be increased [118].

### The transdisciplinary approach to PV second-line therapy

Five years after diagnosis, patient reported progressive aquagenic pruritus and splenomegaly, with spleen palpable at 6 cm below costal margin. Bone marrow biopsy was performed, to assess disease evolution. Bone marrow histology confirmed the setting of polycythemia vera as no fibrotic evolution was detected. NGS was also performed and revealed no additional mutations. She was then evaluated for a switch to ruxolitinib and started the therapy at the standard dose of 10 mg twice-daily. Pre-treatment assessment and monitoring involved a transdisciplinary team:

- Infectious disease specialist

Screening for B and C hepatitis, and for Human immunodeficiency virus was negative. Vaccinations against Varicella Zoster virus (recombinant, adjuvanted), *Haemophilus influenzae*, *Pneumococcus* and *Meningococcus* were recommended and subsequently administered. Although these vaccinations are generally performed through community-based vaccination programs, an ideal care pathway would include the possibility of administering them directly in the hospital setting. Since Quantiferon test was positive, with negative chest X-ray, isoniazid prophylaxis was administered for 6 months.

- Hospital pharmacist

Ruxolitinib re-supply involved the local hospital pharmacist, who verified the prescription and monitored adherence alongside the hematologist. The RAMP study prospectively evaluated the ARMS-12 [119], a validated tool for assessing treatment adherence, alongside the Distress Thermometer [78], in a cohort of MPN patients over 48 weeks of ruxolitinib treatment. The study revealed suboptimal adherence and high psychological distress in MF and PV patients, mainly related to individual patient characteristics and treatment duration; male patients and those on ruxolitinib for > 1 year were at higher risk. Additionally, logistical challenges within the re-supply system emerged as significant barriers. These findings reveal an unmet clinical need requiring a multifaceted approach, that considers gender, health literacy, symptom burden, disease subtype, and treatment duration. Accurate assessment of adherence may be increasingly relevant in clinical practice, and strategies should be implemented to address both systemic and organizational barriers, as well as to enhance patient awareness and engagement [120]. In this context, the ARMS-7 scale may offer a more practical and time-efficient alternative for use in real-world settings [121]. The patient was monitored in collaboration with:

- Cardiologist and nutritionist for weight gain, hyperlipemia and hypertension to minimize iatrogenic vascular risk. Ruxolitinib treatment has been associated with increases in body weight, systemic blood pressure, cholesterol and triglycerides levels, metabolic changes that may contribute to elevated cardiovascular risk [122, 123]. Regular monitoring and appropriate preventive strategies are therefore essential during therapy.
- Dermatologist for regular visits to detect NMSCs early. Although cases of NMSCs have been reported in patients treated with ruxolitinib, current evidence does not support a causal relationship between ruxolitinib use and the development of NMSCs. In the ruxolitinib global safety database, the incidence of NMSC was 0.46 cases per 100 patient-year. Risk factors observed among patients who developed NMSC include prolonged exposure, male sex, older age and prior skin cancer history and/or HU therapy. Therefore, while appropriate dermatologic monitoring remains advisable, a direct association between ruxolitinib and increased NMSC risk has not been observed [38, 124–126].
- Specialist in thrombosis and hemostasis for ongoing DOAC therapy, with referrals as needed.

At the latest contact, the patient remained asymptomatic, on stable ruxolitinib, with > 50% spleen volume reduction at 6 months (palpation and imaging) and optimal disease control.

## Clinical case 2 - revising the definition of “low-risk” in PV

A 36-year-old woman complaining of itching, fatigue, and headache was diagnosed with PV. She was a mild smoker, with no relevant personal or family history, and had had an uneventful pregnancy three years earlier. No thrombotic events were reported.

Blood counts showed: Hb, 18.5 g/dL; hematocrit, 55.5%; MCV 81 fL; WBC count, 10.8 × 10^9^/L; neutrophils 8.1 × 10^9^/L, lymphocytes 1.5 (NLR 5.4); platelets, 400 × 10^9^/L. EPO levels were suppressed (< 2.5 mU) and ferritin low (10 ng/mL); metabolic profile was normal.

*JAK2*^V617F^ mutation was detected (VAF 45%). Spleen was non palpable, and abdominal US confirmed normal spleen/liver size with preserved splanchnic flow.

The diagnosis was communicated with a distress score of 4 on the distress thermometer [78]. Psychological support was offered but declined. Smoking cessation was strongly recommended, and low-dose aspirin plus phlebotomy were started.

Target hematocrit < 45% was achieved after 8 weeks; however, phlebotomies remained necessary every 6–8 weeks (approximately 7 per annum) over the following 2 years. The hematologist was contacted by the transfusionist regarding the patient’s clinical worsening, characterized by persistent headache, major fatigue, hair loss and angular cheilitis, impairing daily functioning and self-perception. For the first time the patient felt ill, with consequent mood alteration, apathy, and need for psychological support. Blood counts revealed: Hb 14.5 g/dL; hematocrit 47.6%, MCV 70 fL; WBC 17.4 × 10^9^; neutrophils 13.1 × 10^9^/L; platelets 678 × 10^9^; ferritin < 3 ng/L. The patient required frequent phlebotomies and exhibited persistent leukocytosis, iron deficiency, and high symptom burden, which was assessed by the MPN10-Total Symptoms Score (TSS: 34) [127].

### The evolving concept of low-risk PV

Traditionally, cytoreduction in PV is reserved to HR patients. However, the definition of LR disease is evolving, driven by novel agents, improved detection of patients who could benefit from treatment intensification, and recognition that LR PV carries more than twice the thrombotic risk of the general population. Increasing evidence suggests that age > 60 and prior thrombosis only partially capture the overall risk profile of PV, prompting the search for additional biomarkers to refine stratification. Since many LR display significant symptoms, the European LeukemiaNet (ELN) and literature advocate for cytoreduction in LR patients presenting specific clinical signs and symptoms (CSSs) (Table 2) [128].

**Table 2:** Indications to cytoreduction in polycythemia vera

High Risk patients	Low Risk patients		
Cytoreduction mandatory	Cytoreduction recommended	Cytoreduction should be considered	Trial or cytoreduction can be considered
• Age ≥ 60 years and/or • Previous thrombosis	• Poor tolerance to phlebotomy (recurrent syncopes or blood phobia or severe difficulties in venous access); • Symptomatic progressive splenomegaly (increase by > 5 cm in the last year); • Persistent leukocytosis (WBC > 20 × 10^9^/L) for 3 months	• Progressive/Persistent leukocytosis (100% increase if WBC < 10 × 10^9^/L; 50% increase if WBC > 10 × 10^9^/L; WBC > 15 for more than 3 months • Extreme thrombocytosis (> 1500 × 10^9^/L); • Inadequate hematocrit control with phlebotomies (need for at least 6 phlebotomies per year for at least two years)	• High symptom burden (TSS ≥ 20) or severe itching (itching score ≥ 5) that are not ameliorate by phlebotomy, antiplatelet therapy or antihistamine; • Relevant cardiovascular risk; • High JAK2 VAF; • High absolute neutrophil count

ELN criteria for therapy start (strength of the recommendation: weak)

*ELN* European LeukemiaNet, *JAK2* Janus Kinase 2, *TSS* Total Symptom Score, *VAF* Variant Allele Frequency, *WBC* White Blood Cells

In this case, the patient developed progressive leukocytosis (50% increase from baseline count that was > 10 × 10⁹/L), poor hematocrit control despite phlebotomy, and a remarkable symptom burden (TSS ≥ 20).

Among PV patients uniformly treated with first-line HU, those with CSSs had a significantly higher thrombotic risk: IRR 2.2 vs. 0.7 per 100 PY (*p* < 0.001) and 5-year TFS 88.7% vs. 96.1% (*p* < 0.001). The best TFS was observed in LR and HR-AGE patients without CSSs (100% and 98.1%, respectively). LR and HR-AGE patients with CSSs, as well as HR-THRO patients without CSSs, had similar outcomes (~ 89–92%), whereas HR-THRO remained the highest risk group (5-year TFS 80.2%). In multivariate analysis, inadequate Hct control, progressive splenomegaly, CVRFs, and prior thrombosis independently predicted thrombosis. Overall, CSSs were common and identified patients at higher risk within each category [11].

Beyond CSSs, novel factors are being investigated. A French group proposed a 4-item score, the ARterial Thrombosis Score (ARTS), including arterial thrombosis, age > 60 years, CVRFs, and presence of *TET2* or *DNMT3A* mutations. ARTS performed better than conventional 2-tiered risk stratification in identifying LR and HR patients, with an event rate of 0.37% vs. 1.19% patients-year, respectively [129]. *JAK2* allele burden has been linked to increased thrombotic risk and a VAF cut-off > 50% was seen to be an independent risk factor for venous thrombosis, especially in LR PV [130, 131]. An NLR ≥ 5 is common in older PV patients with more CV risk factors, more arterial events, and more aggressive blood counts, reflecting a more proliferative phenotype. An NLR ≥ 5 also predicted poorer overall survival, with more than double the mortality compared with NLR < 5. These findings were confirmed in a Danish MPN cohort, in which a higher NLR was similarly associated with increased all-cause mortality [132, 133]. Finally, three-tiered mutation-enhanced international prognostic systems (MIPSS) have been developed and validated in cohorts of ET and PV patients, showing a superiority vs. conventional scoring systems in survival prediction, with particular reference to spliceosome mutations [134].

### Proposal of a novel cytoreductive treatment algorithm

**Fig. 4:**
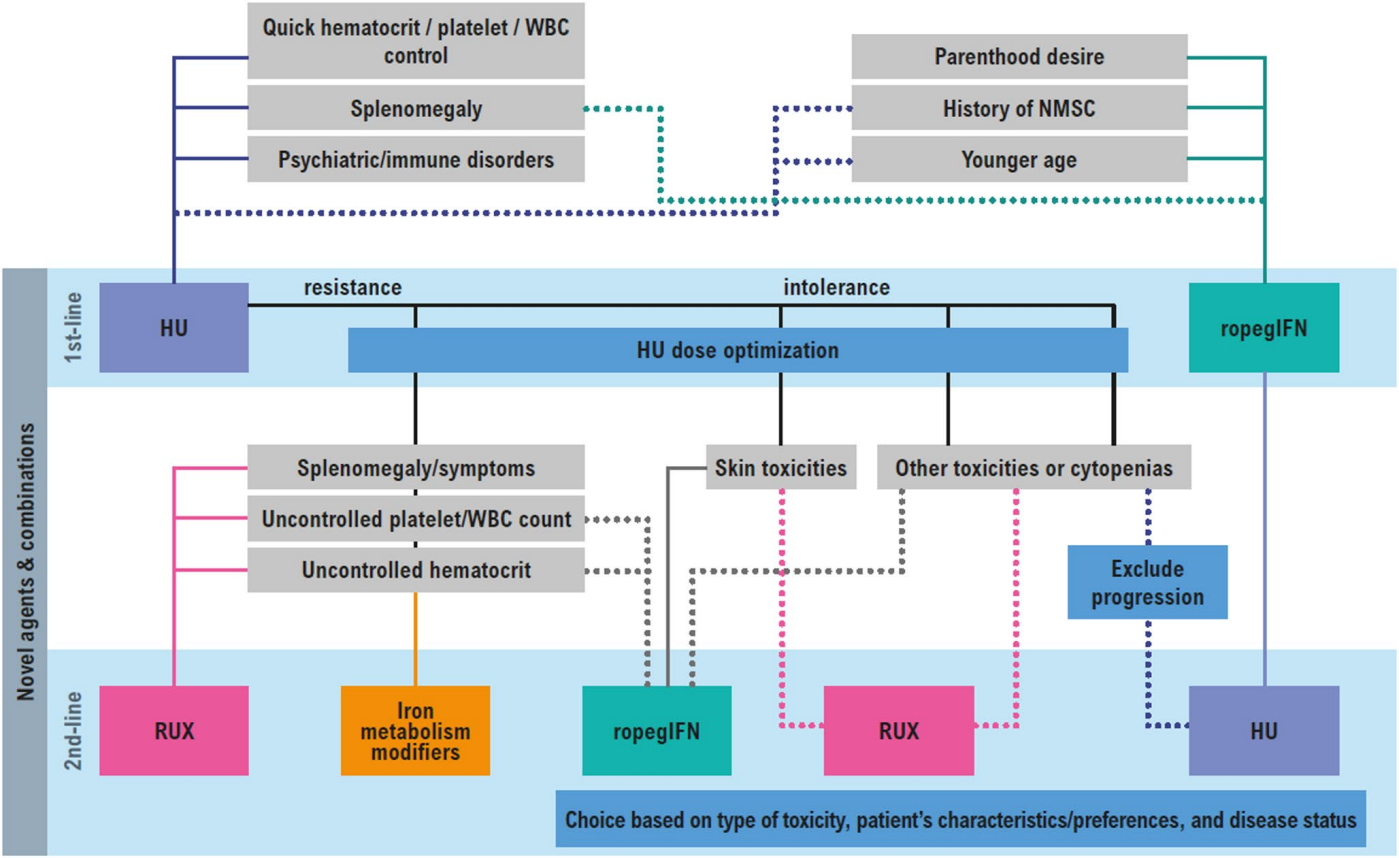
Proposal of a cytoreductive treatment algorithm in polycythemia vera where treatment choice is based on type of toxicity, patient’s characteristics/preferences, and disease status. Abbreviations: HU: Hydroxyurea; NMSC: Non-melanoma skin cancer; RopegIFN: Ropeginterferon; RUX: Ruxolitinib; WBC: White blood cell

Based on all the emerging evidence suggesting that conventional criteria may reflect a limited view of the overall risk profile, and in light of a transdisciplinary, patient-centered approach, we propose a novel cytoreductive treatment algorithm that considers the patient’s characteristics/preferences, the type of treatment’s toxicity, and the disease status, rather than the established risk factors (Fig. 4).

### Transdisciplinary approach in a low-risk PV patient

The recognition of clinical signs and symptoms promoting cytoreductive therapy required a multidisciplinary approach.

- Hematologists and transfusion medicine specialist
  Assessing symptom burden in PV can be challenging. Common symptoms include fatigue, aquagenic pruritus, early satiety, headache, weight loss, and night sweats, often worsened by iron deficiency from frequent phlebotomies. Validated tools such as the Myeloproliferative Neoplasm Symptom Assessment Form allow more objective evaluation [127]. Close collaboration with transfusionists helps monitor phlebotomy frequency and tolerance, enabling early detection of clinical worsening, as in this case.

- Onco-psychologist
  MPNs profoundly affect daily life, causing anxiety, depression, and work-related limitations [135–138]. Psychological support should therefore be considered when the DT score is > 5.

Thyroid screening prior to cytoreduction was negative. The presence of thyroid autoantibodies (TPO and thyroglobulin) significantly raises the risk of autoimmune thyroid disease, especially Hashimoto’s thyroiditis. While predictive, these antibodies alone—without dysfunction—do not contraindicate IFN therapy but require close endocrinological follow-up. Autoantibodies may also develop during treatment, leading to hypothyroidism, which can be subclinical (elevated TSH, normal fT4) or overt (elevated TSH, low fT4). Levothyroxine is indicated for overt cases and considered in subclinical ones with TSH > 7 mU/L or rising levels and high anti-TPO titers. Hypothyroidism often persists despite stopping IFN, so therapy can usually continue with hormone replacement. Hyperthyroidism is rare but may indicate destructive thyroiditis or Graves’ disease, requiring urgent endocrine evaluation and IFN discontinuation [139].

Conversely, autoimmune screening resulted positive for Rheumatoid Factor (RF) and Anti–citrullinated protein antibody (ACPA). The distress score increased to 8. The patient was referred to:

- a dedicated psychologist for evaluation for mood disturbance. Mood alterations were described as reactive to the clinical condition and possibly beneficial to treatment initiation, with psychological monitoring.
- a rheumatologist. In absence of clinical signs of rheumatological disease, a twice-a-year follow-up was established, with no contraindications to interferon-based therapies. Notably, the management of rheumatologic diseases during interferon therapy requires careful baseline evaluation and individualized risk assessment. Prior to initiating treatment, patients should undergo a Complete Blood Count, biochemistry panel, and acquisition of autoimmune profile is useful to monitor and intercept disimmune alterations. For those with connective tissue diseases, additional tests such as Complement 3 (C3), Complement 4 (C4), and 24-hour proteinuria are recommended. Mild forms of rheumatoid arthritis, spondylarthritis, psoriatic arthritis, or undifferentiated connective tissue disease that do not require immunosuppressive therapy are not absolute contraindications to interferon use, though decisions should be made collaboratively with a hematologist. Conversely, patients receiving immunosuppressive agents like Methotrexate, Cyclosporin A, Leflunomide, or biologics generally present a contraindication to interferon-based therapies, with monitoring and management tailored to individual clinical needs [71, 72].

With no suitable clinical trial available, ropegIFNα2b was initiated to control symptoms and myeloproliferation. After 6 months, phlebotomies were no longer required, symptom burden improved, and iron deficiency-related symptoms resolved. Mood monitoring was positive and no immune adverse findings were appreciated.

In case of pregnancy desire, the patient was advised to promptly inform the hematologist and continue treatment with ropegIFNα2b, which is considered feasible during both pregnancy and lactation. A multidisciplinary approach would also be activated, involving expert gynecologists and obstetricians to ensure coordinated and individualized care. Pregnancy in MPN patient may be burdened by higher complication rates but should not be discouraged [107, 140, 141].

## PV transdiciplinary management: how we do it

These two clinical cases aim to summarize some of the players involved in transdisciplinary patient care. Not all patients require referrals to every specialist. Nevertheless, we have established a medical team with dedicated specialists to ensure timely referral of each patient to the appropriate healthcare professional according to their clinical needs. All patients require the medical professionals indicated in Fig. 2. The closest collaboration is between the hematologist and the clinical research nurse (Fig. 2S), who work side by side during outpatient visits. Referral to other specialists and the intensity of the care settings depend on the patients’ risk profile, and are organized as follows:

### Transfusion medicine specialist

Proximity to the patient’s residence is a key factor for adherence to therapeutic phlebotomy. The general practitioner refers the patient to the transfusion center closest to their home. In the case of a patient living near the referral hospital for the treatment of polycythemia vera, the hematologist refers the patient to the hospital-based transfusion center.

- Cardiologist
  A baseline cardiovascular risk assessment - including blood glucose, lipid profile, ECG, and carotid artery ultrasound - is prescribed bythe hematologist for all patients. Subsequently, patients undergo an initial evaluation by a dedicated cardiologist through direct referraland dedicated appointment slots for PV patients. In low-risk patients, subsequent follow-up is performed by a community cardiologist.Conversely, in high-risk patients, long-term monitoring and management are provided by a hospital-based cardiologist.
- Infectious disease specialist
  We recommend seasonal vaccination influenza against COVID for all patients, usually administered by their general practitioner. In those with a prior history of herpes zoster (HZ) vaccination with the adjuvanted recombinant zoster vaccine (aRZV) is advised regardless of age or ongoing therapy. Pneumococcal vaccination is also recommended in patients over 65 years or with comorbidities such as COPD or cardiovascular disease.
  Before starting ruxolitinib, baseline screening includes hepatitis B and C, HIV, and tuberculosis infection testing; patients with Tuberculosis infection or occult HBV infection are referred to infectious disease specialists for appropriate management. We strongly recommend aRZV prior to ruxolitinib and HBV vaccination if not previously performed. Because HZ risk persists over time and no trials directly compare vaccination with antiviral prophylaxis, we favor vaccination, with oral antiviral prophylaxis reserved for patients with previous HZ at higher risk of reactivation [142]. An Italian consensus (2023) proposed early aRZV with short-term antiviral prophylaxis after the second dose [143]. Additional vaccinations against pneumococcus and other encapsulated bacteria may be appropriate based on comorbidities [144, 145].
  To improve access, we have established a dedicated hospital–community pathway led by infectious disease specialists, who evaluate individual risk and administer tailored vaccination schedules. Despite chronic inflammation and immune dysfunction in MPNs, exacerbated by cytoreductive and immunosuppressive therapies, vaccination remains strongly recommended, ideally before ruxolitinib initiation [146–148]
- Radiologist
  We prescribe an annual abdominal ultrasound for all patients, which is performed in community facilities. However, in patients with abnormalities of the splenic–portal flow or a history of splanchnic thrombosis, ultrasound assessment is centralized at the hospital’s dedicated imaging service. Conversely, multidisciplinary management in specific clinical contexts (Fig. 3) involves hospital-based specialists, as specific expertise in PV is required. The only exception is dermatologic evaluation, which is managed in community settings in patients without a history of NMSCs.
  Patients are referred by the hematologist to the appropriate specialists through a dedicated internal visit reservation channel. In this high‑intensity care setting, the patient’s management is reviewed in multidisciplinary meetings to evaluate treatment modifications and major clinical events (i.e., pregnancy, recurrent thrombosis).

## PV management: innovations beyond conventional cytoreduction

Clinical trials in PV are essential for advancing therapeutic strategies, improving symptom control, and addressing unmet needs in disease monitoring and risk stratification. Research efforts are shifting from hematocrit and symptoms control toward broader disease modification. Clonal suppression, assessable via peripheral *JAK2*^V617F^ VAF monitoring, is emerging as a key target. Trials suggest that reducing VAF may improve blood counts and reduce thrombotic and progression risk both with ropegIFNα2b and ruxolitinib.

However, participation in clinical trials requires significant time and resources from both healthcare system and patients/caregivers, potentially contributing to indirect social costs [149, 150]. In this context, the presence of a dedicated clinical research nurse becomes crucial–not only to coordinate study procedures and ensure protocol adherence–but also to support patient engagement, streamline communication, and safeguard continuity of care throughout the trial process [151, 152]. Their clinical expertise and patient-centered approach enhance both the scientific integrity of the research and the overall patient experience, making them an indispensable asset in the success of clinical trials (Fig. 2).

In Table 3, we summarize ongoing and recent trials (January 2024 - June 2025) exploring strategies beyond hematocrit control.

**Table 3:** Summary of selected ongoing phase 1 or later clinical trials for polycythemia vera treatment

Drug name	Mechanism of action	Official study title and ID	Trial current status	Primary outcome measure(s)
IFN-based				
Ropeginterferon alfa-2b	Modulation of immune system	Efficacy and Safety of Ropeginterferon Alfa 2b (P1101) for Patients With Polycythemia Vera – A Randomized Open Label Global Multicenter Study (PARADIGM-PV) (NCT06290765)	Not yet recruiting	The proportion of patients whose hematocrit is maintained without phlebotomy eligibility from Week 20 through Week 32
Ropeginterferon alfa-2b	Modulation of immune system	A Phase IIIb, Randomized, Open-Label, Parallel Group, Multicenter Study to Assess Efficacy, Safety, and Tolerability of Two Dosing Regimens of Ropeginterferon Alfa-2b-njft (P1101) in Adult Patients With Polycythemia Vera (PV) (NCT05481151)	Active, not recruiting	Compare efficacy, safety, and tolerability of P1101 utilizing 250-350-500 mcg compared to the current labeled dosing through assessing the proportion of subjects that are in a complete hematologic response at Week 24
Ropeginterferon alfa-2b	Modulation of immune system	A Phase II Single-Arm Multicenter Study to Assess Efficacy and Safety of P1101 in Chinese Polycythemia Vera Patients Who Are Intolerant or Resistant to Hydroxyurea (NCT05485948)	Active, not recruiting	The phlebotomy- or erythrocytapheresis-free CHR rate based on the central laboratory test results evaluation
Ropeginterferon alfa-2b	Modulation of immune system	LOW-PV Continuation (NCT06752941)	Active, not recruiting	Maintenance of Treatment response in patients enrolled in the LOW-PV RCT study who continued to receive ropeginterferon alfa-2b until the conclusion of the study on March 31, 2023
JAK inhibition				
Ruxolitinib	JAK inhibitor	A Phase 2 Study Of Ruxolitinib In Low-Risk Essential Thrombocythemia And Polycythemia Vera With Significant Symptom Burden (NCT04644211)	Recruiting	Percentage of patients who achieve > 50% reduction from baseline to Myeloproliferative Neoplasm Symptom Assessment Total Symptom Score
Ruxolitinib	JAK inhibitor	A Phase III, Randomised, Open-label, Multicenter International Trial Comparing Ruxolitinib With Either HydRoxycarbamIDe or Interferon Alpha as First Line ThErapy for High Risk Polycythemia Vera (NCT04116502)	Recruiting	Event Free Survival
Methotrexate	Type 2 JAK inhibitor	MethoTRExATE in MyelOpRolifErative Neoplasms (TREATMORE) Trial (NCT06541249)	Recruiting	Myelofibrosis overall response rate; Polycythemia vera and essential thrombocythemia overall response rate
Iron metabolism modifiers				
Rusfertide	Hepcidin mimetic	An Extension Study to Evaluate the Long-term Safety of Rusfertide (PTG-300) in Subjects With Polycythemia Vera (THRIVE) (NCT06033586)	Enrolling by invitation	Hematocrit; Phlebotomies
9MW3011	anti-TMPRSS6 monoclonal antibody	A Phase Ib, Multicenter, Randomized, Open-Label, Dose Escalation Study to Evaluate the Safety, Tolerability, Pharmacokinetics, Pharmacodynamics, and Immunogenicity of 9MW3011 in Patients With Polycythemia Vera (NCT06752746)	Recruiting	Adverse Event; Vital sign; Physical examination; 12-lead electrocardiogram; Laboratory test result
BEBT-507	TMPRSS6-targeted siRNA	A Multicenter, Open-Label Phase I Clinical Trial of BEBT-507 Injection in Subjects With Polycythemia Vera (PV) (NCT07012109)	Not yet recruiting	Maximum Tolerated Dose; Dose-Limiting Toxicity; Proportion of subjects with hematocrit < 45% without or with specified therapies (phlebotomy or erythrocytapheresis)
SLN124	TMPRSS6-targeted siRNA	Phase 1/2 Study With an Open-label Dose Escalation Phase Followed by a Randomized, Double-blind Phase of SLN124 in Patients With Polycythemia Vera (NCT05499013)	Recruiting	Incidence of treatment-emergent adverse events; Assessment of the number of phlebotomies at intervals
DISC-3405	anti-TMPRSS6 monoclonal antibody	A Phase 2, Open-Label Study of the Safety, Tolerability, Pharmacokinetics, Pharmacodynamics, and Efficacy of DISC-3405 in Participants With Polycythemia Vera (PV) (NCT06985147)	Not yet recruiting	Number of participants with treatment-related adverse events as assessed by CTCAE; Incidence of clinically abnormal vital signs; Incidence of clinically abnormal physical exam; Incidence of clinically abnormal electrocardiograms; Incidence of abnormal laboratory test results;
Cytoreductive agents with a novel mechanism of action				
Givinostat	HDAC inhibitor	Randomized, Open-label, Multicenter Phase 3 Study to Assess the Efficacy and Safety of GIVinostat Versus Hydroxyurea IN JAK2V617F-positive High-risk Polycythemia Vera Patients: the GIV-IN PV TRIAL (NCT06093672)	Recruiting	Proportion of patients achieving a response at Week 48
Bomedemstat	LSD1 inhibitor	Investigator-Initiated Trial of the LSD1 Inhibitor IMG-7289 for the Treatment of Patients with Essential Thrombocythemia (ET) or Polycythemia Vera (PV) That Have Failed At Least One Standard Therapy (NCT04262141)	Active, not recruiting	Hematologic Response Rates
Bomedemstat	LSD1 inhibitor	A Multicenter, Open-Label, Extension Study Evaluating the Safety and Efficacy of Bomedemstat for the Treatment of Participants Enrolled in a Prior Bomedemstat Clinical Study (NCT06351631)	Recruiting	Percentage of participants with one or more adverse events; Percentage of participants who discontinued study treatment due to an adverse event

## Conclusion

PV may manifest decades after initial driver mutations and clonal HSCs expansion, offering a potential window for early screening, diagnosis, and intervention before thrombotic events. Emerging therapies across diverse drug classes reflect a shift toward broader disease modification. Molecular profiling could further refine therapeutic selection and response monitoring [2], reinforcing the emerging cytoreductive algorithm. Crucially, this paradigm calls for a transdisciplinary approach, where healthcare professionals collaborate beyond disciplinary boundaries to co-manage the biological, emotional, and systemic dimensions of PV. This integrated care enhances continuity, anticipates patient frailty, and supports individualized, proactive disease management.

While this narrative review synthesizes current evidence and highlights evolving multidisciplinary strategies in PV management, it is limited by its non-systematic nature and the heterogeneity of available studies, underscoring the need for further prospective research to validate these approaches.

## Supplementary Information

Supplementary Material 1. Figure 1S Evolution from multidisciplinary to interdisciplinary and transdisciplinary models of care. Figure 2S Structure of a transdisciplinary care model for patients with polycythemia vera. Abbreviations: NMSCs: Non-melanoma skin cancers.

## Data Availability

No datasets were generated or analyzed during the current study.
