# Supplementary material for: Modern and multidisciplinary care in polycythemia vera
Source: Ann Hematol. 2026 Apr 18;105(5):251. doi: 10.1007/s00277-026-06970-w (PMC13091898; doi:10.1007/s00277-026-06970-w)
Supplement: Supplementary file 1 — Supplementary Material 1. Figure 1S Evolution from multidisciplinary to interdisciplinary and transdisciplinary models of care. Figure 2S Structure of a transdisciplinary care model for patients with polycythemia vera. Abbreviations: NMSCs: Non-melanoma skin cancers. [file 277_2026_6970_MOESM1_ESM.docx]

**Figure 1S** Evolution from multidisciplinary to interdisciplinary and transdisciplinary models of care

**
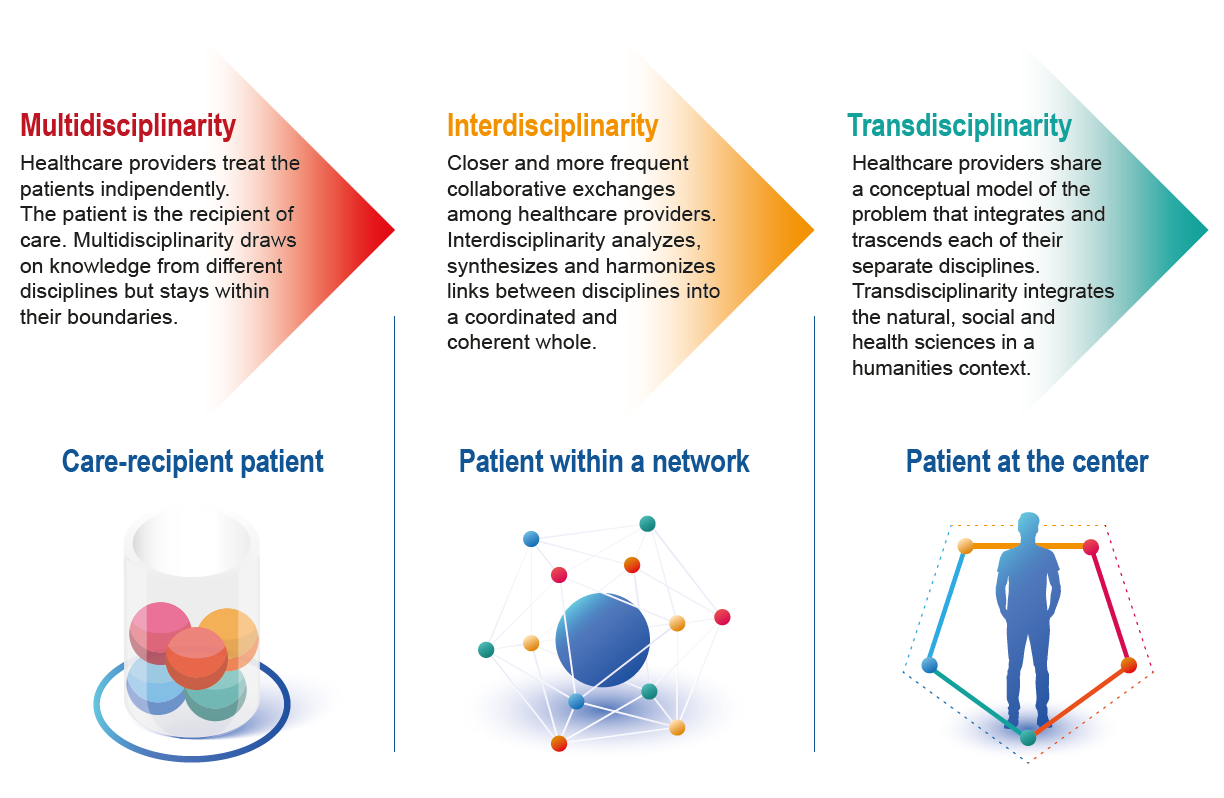
**

**Figure 2S** Structure of a transdisciplinary care model for patients with polycythemia vera

**
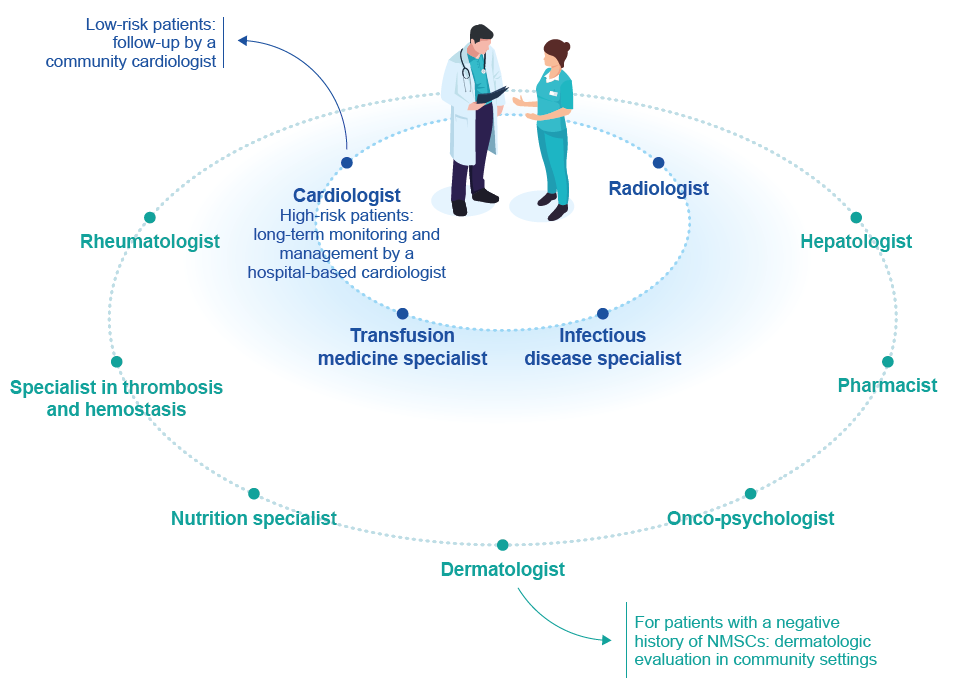
**

**Abbreviations**: NMSCs: Non-melanoma skin cancers
